# Integration of Three‐Dimensional Cell Culture Techniques and Photobiomodulation for Supportive Differentiation of Adipose‐Derived Stem Cells into Smooth Muscle

**DOI:** 10.1002/cbf.70250

**Published:** 2026-06-30

**Authors:** Christevie Mbuyu, Heidi Abrahamse, Anine Crous

**Affiliations:** ^1^ Laser Research Centre, Faculty of Health Sciences University of Johannesburg Doornfontein Johannesburg South Africa

**Keywords:** photobiomodulation, smooth muscle differentiation, stem cells, three‐dimensional hydrogel culture, tissue engineering

## Abstract

Photobiomodulation (PBM) has gained recognition as a promising, non‐invasive strategy to enhance the differentiation of adipose‐derived stem cells (ADSCs) into smooth muscle cells (SMCs), particularly when used together with a three‐dimensional (3D) hydrogel culture system. This review explores the key mechanisms by which PBM modulates cellular behavior, along with its role in the promotion of SMCs lineage commitment in ADSCs. We investigate the influence of various light parameters including wavelength, intensity, energy density, exposure time and duration on cell responses such as proliferation, metabolic activity, gene expression and functional maturation. Moreover, we scrutinize the different hydrogel scaffolds, their compositions and the respective effects on PBM efficacy and consequential cellular outcomes. We also highlight the importance of scaffold design in mimicking the native extracellular matrix and how along with certain mechanical cues, they play a vital role in supporting stem cell behavior. By identifying current challenges, gaps in the literature and proposing future recommendations; this review aims to provide insights into the optimization of PBM protocols to improve SMCs differentiation of ADSCs, ultimately with the intention of integrating PBM to optimize regenerative therapies for use in regenerative medicine and tissue engineering (TE) strategies for vascular conditions and other smooth muscle‐related diseases.

## Summary

This review integrates photobiomodulation (PBM) and three‐dimensional (3D) hydrogel systems as complementary regulators of adipose‐derived stem cell differentiation toward smooth muscle lineages, an approach rarely examined in combination. It synthesizes evidence on light‐mediated modulation of cellular metabolism, mechano‐transduction, and lineage‐specific signaling within biomimetic 3D matrices, providing a cohesive framework for understanding how biophysical and biochemical cues converge to direct smooth muscle formation. Key hydrogel properties, matrix design considerations, and PBM parameters essential for functional tissue development are critically discussed, alongside current biological and translational challenges. Given the importance of smooth muscle in vascular and urological regeneration, this review offers timely insight for advancing stem cell‐based tissue engineering strategies, aligning closely with the focus of Cell Biochemistry and Function on cellular function and regenerative biomaterials.

**Abbreviations**
3D: three‐dimensional
ADSCs: adipose‐derived stem cells
APC: adenomatous polyposis coli
ATP: adenosine triphosphate
CArG: CC(A/T‐rich)6GG element
CCO: cytochrome c oxidase
CK1: casein kinase 1
ECM: extracellular matrix
ERK: extracellular‐regulated kinase
FDA: food and drug administration
FOXO: Forkhead box O
FOXO1: Forkhead box O1
FOXO4: Forkhead box O4
GSK‐3 β: glycogen synthase kinase 3 β
HDAC: histone deacetylases
IL‐6: interleukin‐6
LRP‐5/6: low‐density lipoprotein receptor‐related proteins 5 and 6
MAP2K: MAPK kinase
MAP3K: MAPK kinase kinase
MAPK: mitogen activated protein kinase
MEK: mitogen activated protein kinase
MMP: Mitochondrial membrane potential
MSC: mesenchymal stem cells
mTORC2: mammalian target of rapamycin complex 2
NF‐κB: nuclear factor kappa B
NO: nitric oxide
PBM: photobiomodulation
PDK1: 3‐phosphoinositide‐dependent protein kinase 1
PEG: polyethylene glycol
PI3K/Akt: phosphatidylinositol‐4,5‐bisphosphate 3‐kinase/protein kinase B
PI(3,4,5)P3: phosphatidylinositol (3,4,5)‐trisphosphate
PI(4,5)P2: phosphatidylinositol 4,5‐bisphosphate
RA: retinoic acid
Raf: rapidly accelerated fibrosarcoma
Ras: rat sarcoma
RGD: arginylglycylaspartic acid
ROS: reactive oxygen species
Ser473: serine 473
SM22α: smooth muscle protein 22 alpha
SMAD: Suppressor of Mothers against Decapentaplegic
SMCs: smooth muscle cells
SMMHC: smooth muscle myosin heavy chain
SMαA: smooth muscle α actin
SRF: serum response factor
TE: tissue engineering
TGFβ: transforming growth factor β
TGFβR1: transforming growth factor β receptor 1
TGFβR2: transforming growth factor β receptor 2
Thr308: threonine 308
TNF‐α: tumor necrosis factor α
Wnt: wingless‐related integration site

## 1 Introduction

Smooth muscle refers to an involuntary, non‐striated muscle with neural innervation deriving from the autonomic nervous system [1]. Their contractile function is essential for processes such as angiogenesis, regulation of hollow organ function, and maintenance of blood pressure. However, inflammation, oxidative stress, dedifferentiation and disturbances in extracellular matrix (ECM) remodelling contribute to alterations in smooth muscle cell (SMCs) structure and functions, ultimately affecting the organs where they are found [2, 3, 4].

It is therefore no surprise that there is an increased demand of SMCs for replacement therapy [1]. This approach is pivotal in the treatment of various ailments involved in smooth muscle diseases of various organs, including the respiratory tract, gastrointestinal tract, urinary bladder, vascular system, uterus as well as the male reproductive organs [1]. One of the reported applications of SMCs in regenerative medicine has been in vascular tissue engineering (TE), where the development of artificial blood vessels for coronary bypass surgery and other vascular graft applications relies on the functional integration of SMCs [5]. Furthermore, the ability of the artificial tissue to produce the ECM components allow the imitation of natural blood vessels [6]. Despite these advancements, the generation and sourcing of functionally stable SMCs remains a challenge and major limitation of TE. Cells isolated from tissues often exhibit reduced proliferation capacity and loss of contractile phenotype in vitro, while those obtained from diseased tissues may retain pathological characteristics. To circumvent these challenges, alternative cell sources in the form of ADSC have been explored.

Adipose‐derived stem cells (ADSCs), a type of mesenchymal stem cells (MSCs) found in the stromal‐vascular fracture of adipose tissue have attracted considerable interest in regenerative medicine due to their abundance, ease of harvest and minimal donor site morbidity [7, 8, 9]. They possess significant differentiation and regenerative potential, being multipotent and capable of differentiating into various cell types including adipocytes, osteocytes, chondrocytes and SMCs making them suitable for tissue repair, regeneration and engineering hollow organs and vessels [1, 10, 11]. They can be harvested using fewer invasive methods when compared to other stem cells thus advantageous as a more practical choice for clinical applications [12, 13]. Although ADSCs have demonstrated considerable promise for smooth muscle regeneration, conventional biochemical induction methods alone may not fully replicate the complex signaling environment required for effective lineage commitment, as stem cell differentiation is regulated by a combination of biochemical, mechanical, and microenvironmental cues rather than soluble factors alone [14]. Consequently, adjunct strategies capable of modulating cellular behavior and enhancing differentiation outcomes have gained increasing attention.

Photobiomodulation (PBM) has emerged as one such strategy due to its ability to modulate cellular metabolism, proliferation, gene expression and tissue regeneration [15]. Currently, it serves as a therapeutic technique used in several medical approaches spanning from wound healing, osteoarthritic treatments and nerve injuries [15]. It makes use of low‐level laser therapy with red or infrared along with lower power outputs when compared to alternative laser treatment methods. In turn, this renders the treatment harmless as a result of the lack in thermal consequence whilst stimulating thermal photochemical reactions which are favorable to cellular metabolic processes [15]. Reports indicate that in vivo MSCs proliferation are stimulated by PBM through the potential changes conveyed by gene expressions and their activations [15].

While PBM has demonstrated the potential to enhance cellular responses, the microenvironment in which these processes occur is likewise a critical determinant of differentiation outcomes. Traditional two‐dimensional cell culture systems, performed in petri dishes, flasks, or multiwell plates, do not accurately represent the in vivo environment, with cells exhibiting altered morphology, proliferation and differentiation [16, 17]. In contrast, three‐dimensional (3D) culture systems, particularly hydrogel scaffolds comprising water‐swollen polymer networks, present as promising platforms for modern cell culture since they imitate salient elements of native ECMs; provide mechanics similar to that of soft tissues and support both cell adhesion and protein sequestration [18]. They are useful since they are able to provide an environment that cells are able to adhere, proliferate and differentiate in [19]. By providing structural and biochemical cues, hydrogels mimic key features of the native ECM, thereby supporting stem cell differentiation and tissue development.

There remains however, a limited understanding of how biophysical stimulation strategies such as PBM interact with 3D culture systems to influence ADSC differentiation into SMCs. Therefore, this review aims to critically evaluate the role of PBM in modulating stem cell behavior such as proliferation, gene expression and differentiation, examine the contribution of hydrogel‐based 3D systems, and explore how PBM and hydrogel‐based 3D culture systems may collectively influence ADSC differentiation and SMC TE outcomes.

## 2 Mechanisms Underlying PBM

### 2.1 Mitochondrial Stimulation

In SMCs differentiation, the mitochondria contribute significantly to regulation of smooth muscle phenotype and differentiation [20]. PBM functions to provide a biological effect primarily through the stimulation of cytochrome c oxidase (CCO), a key component of the mitochondrial respiratory chain [21], however, it may also interact with other cellular targets, including additional chromophores, ion channels, and membrane‐associated receptors [22, 23]. Previous studies have shown that PBM enhanced CCO activity, resulting in increased adenosine triphosphate (ATP) generation. This is facilitated by the photodissociation of nitric oxide (NO) from CCO, which promotes proton gradient formation and subsequently drives ATP synthase activity [21]. These absorption peaks usually occur in the red (600–700 nm) and near‐infrared (760–940 nm) spectrum regions [24]. In addition to modulating mitochondrial activity, PBM has been shown to influence reactive oxygen species (ROS) production, NO release and calcium ion homeostasis [21]; with studies such as Crous et al. [25] demonstrating a positive correlation between ATP levels and ADSC proliferation, indicative of enhanced mitochondrial activity following PBM exposure.

### 2.2 Generation of ROS

Studies reveal that PBM induces a modest increase in mitochondrial ROS as a result of photon absorption [24]. This process triggers the activation of redox‐sensitive transcription factors such as nuclear factor kappa B (NF‐κB), which functions as a cellular redox sensor; this is supported by findings showing that antioxidant treatment inhibits NF‐κB activation at wavelengths of 810 nm [26]. The increased production of ROS may elicit mitochondrial signaling pathways leading to several benefits including cytoprotective, antioxidant and anti‐apoptotic cellular responses. In addition, the photodissociation and subsequent release of NO further contributes to vasodilation, signaling and activation of numerous important cellular pathways [24]. These mechanistic observations are supported by experimental data, as reported by Crous and colleagues (2021), where increased ROS levels in PBM‐treated ADSCs were observed across all wavelengths, correlating with enhanced cell proliferation. Notably, these elevated ROS levels were not detrimental but were associated with increased proliferative capacity and self‐renewal, suggesting a regulatory rather than cytotoxic role of ROS in this case. Together, these findings support the concept that PBM‐induced ROS production functions as a controlled signaling mechanism that promotes cellular activation and may contribute to the priming of stem cells for differentiation [25].

### 2.3 Activation of Cell Signaling Pathways

PBM is important in its activation of signaling pathways which are crucial in regulation of several factors required for differentiation. Table 1 summarizes key PBM‐activated signaling pathways relevant to SMCs differentiation, highlighting their functional roles and associated molecular markers.

**Table 1:** PBM‐activated signalling pathways relevant to smooth muscle cell differentiation.

Signaling pathway activated by PBM	Function in SMCs Differentiation	References
Mitogen‐activated protein kinase/extracellular signal‐regulated kinase (MAPK/ERK) pathway	A family of serine protein kinases involved in regulating proliferation, differentiation and apoptosis. Activated through the Ras‐Raf‐MEK‐ERK signaling cascade (MAP3K‐MAP2K‐MAPK), resulting in transcriptional regulation of genes associated with cell fate determination. May interact with TGF‐β signaling through transforming growth factor receptor 1 (TGFβR1)‐mediated activation, suggesting potential crosstalk between MAPK/ERK and TGF‐β pathways during PBM‐mediated cellular responses. Contributes to SMCs differentiation through regulation of proliferation and cytoskeletal remodeling.	Serrage et al. [27, 28]
Wnt/β‐Catenin Pathway	Regulation of cell fate, morphology and proliferation through inhibition of apoptosis. SMC differentiation involves canonical Wnt signalling through Wnt3a and β‐catenin. Leads to increased smooth muscle protein 22 α (SM22α) expression. Activated β‐catenin stimulates MSC differentiation. Wnt is involved in lineage commitment.	[29, 30]
Transforming growth factor‐β (TGF‐β) signaling pathway	Stimulates ECM‐mediated collagen production whilst inhibiting matrix metalloproteinases degradation. Stimulates migration and proliferation. Promotes contractile phenotype of adult SMCs through SMAD pathway. Increases expression of smooth muscle α actin (SMαA), calponin and smooth muscle myosin heavy chain (SMMHC). May interact with other pathways such as MAPK/ERK to mediate PBM‐induced effects.	[26, 27, 28, 30, 31, 32]
Phosphatidylinositol 3‐kinase/protein kinase B (PI3K/Akt pathway)	Allows for cell survival, growth, proliferation, migration, angiogenesis and regeneration. Akt activation inhibits Forkhead Box O1 (FOXO1), required for apoptosis and redox balance maintenance. PI3K/Akt advocates for SMCs stimulation, at least partly by stimulating FOXO4. FOXO4 stimulated nuclear exportation; in turn releasing myocardin (a transcriptional coactivator of smooth muscle genes). FOXO Phosphorylation by Akt is responsible for nuclear exclusion and consequent inhibition.	[33, 34, 35]

**Figure 1:**
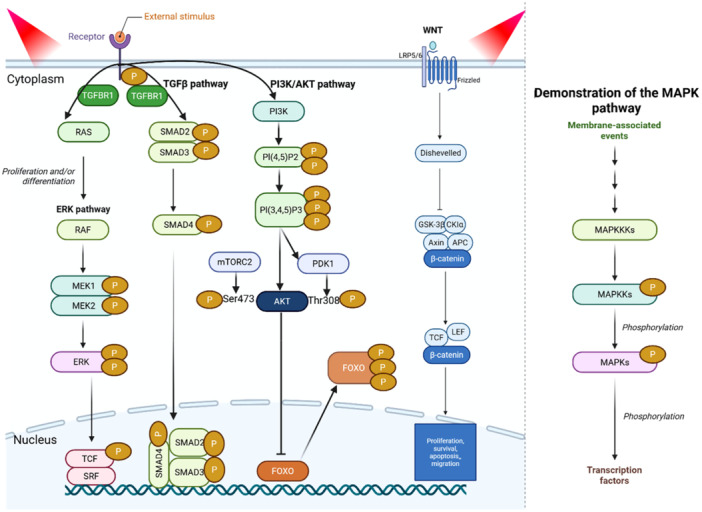
Diagram representing the different SMCs signaling pathways activated by PBM. MAPK/ERK pathway: Activated via MAP3K, which phosphorylates MAP2K, leading to activation of MAPK/ERK signaling and subsequent regulation of gene transcription, proliferation, differentiation and apoptosis; this pathway may be regulated by TGFβR1 [27]). TGFβ signaling: TGFβ (external stimulus) binds TGFβR2/TGFβR1, activating Smad2/3, which complexes with smad4 to induce transcription [36]. PI3K/Akt pathway: PI3K phosphorylates PI(4,5)P_2_ to PI(3,4,5)P_3_, recruiting PDK1, which phosphorylates Akt at Thr308; additional phosphorylation at Ser473 by mTORC2 fully activates Akt, leading to FOXO phosphorylation and nuclear export [37]. Canonical Wnt Pathway: this pathway is β‐catenin dependent, here Wnt proteins activate signaling by binding to Frizzled and LRP‐5/6 receptors, consequently triggering and engaging the Axin complex to the receptor [38]. This complex comprises Axin, APC and kinases including the GSK‐3 β and CK1, of which the kinases work to phosphorylate LRP‐5/6, prompting to the internalization of the complex into endosomes and the subsequent formation of multivesicular bodies [38, 39]. Thes bodies allow for the sequestration of GSK‐3 β, allowing for unphosphorylated β‐catenin to accumulate and translocate to the nucleus, driving proliferation and cell fate [38]. (Created with BioRender, Christevie Mbuyu). APC, adenomatous polyposis coli; CK1, casein kinase 1; ERK, extracellular signal‐regulated kinase; FOXO, Forkhead box O; GSK‐3 β, glycogen synthase kinase 3 β; LRP‐5/6, low‐density lipoprotein receptor‐related proteins 5 and 6; MAPK, mitogen‐activated protein kinase; MAP2K, mitogen‐activated protein kinase kinase; MAP3K, mitogen‐activated protein kinase kinase kinase; mTORC2, mammalian target of rapamycin complex 2; MEK, mitogen‐activated protein kinase; PDK1, 3‐phosphoinositide‐dependent protein kinase 1; PI(4,5)P2, phosphatidylinositol 4,5‐bisphosphate; PI(3,4,5)P3, phosphatidylinositol (3,4,5)‐trisphosphate; PI3K/Akt, phosphatidylinositol‐4,5‐bisphosphate 3‐kinase/protein kinase B; SMAD, Suppressor of Mothers against Decapentaplegic; TGFβ, transforming growth factor β; TGFβR1, transforming growth factor β receptor 1; TGFβR2, transforming growth factor β receptor 2; Thr308, threonine 308; Ser473, serine 473; Wnt, wingless‐related integration site.

Signaling pathways activated following PBM irradiation do not function independently but rather form an interconnected regulatory network. As illustrated in Figure 1, activation of TGF‐β signaling may interact with MAPK/ERK pathways involved in cytoskeletal remodeling and lineage commitment, while PI3K/Akt and Wnt/β‐catenin signaling contribute to cellular survival, proliferation and cell fate determination [36]. Collectively, these pathways regulate processes involved in cellular proliferation, survival and smooth muscle biology. While PBM has been reported to modulate several of these signaling pathways, direct evidence linking PBM‐induced pathway activation to ADSC‐to‐SMC differentiation remains to be explored.

### 2.4 Gene Expression Modulation

Gene expression regulation is fundamental to SMCs differentiation, particularly through transcriptional and epigenetic mechanisms governing contractile phenotype development. Histone deacetylases (HDACs) play important roles in chromatin remodeling and regulation of SMCs‐associated gene expression, thereby influencing SMCs lineage commitment [30, 40]. HDACs comprise several phylogenetic classes, including class I (HDAC1, 2, 3, and 8) and class II (HDAC4, 5, 6, 7, 9, and 10). Inhibition of class I HDACs has been associated with reduced expression of important SMCs markers crucial for SMCs contractile properties namely, SMMHC, SMαA, calponin or SM22α [30, 41]. Additionally, HDAC 7 contributes to SMCs differentiation through regulation of serum response factor (SRF) via myocardin binding, promoting interaction of the SRF‐myocardin complex with SM22α and subsequent activation of SMCs‐associated gene expression to drive SMCs differentiation [30, 40]. Myocardin functions as a major SRF coactivator, responsible for the transcriptional activation of smooth muscle‐specific genes. Its role includes enhancing SRF promyogenic activity by means of histone acetylation at SRF‐binding regions, a fundamental step for ADSC differentiation into SMCs [40, 42, 43]. The activation of SMCs‐specific genes by myocardin relies on the interaction with CArG box regions; a cis‐acting DNA sequence located in the regulatory regions of numerous early and SMCs‐specific genes, important for SMCs differentiation [30, 41, 44].

Furthermore, activation of the PI3K/Akt pathway has been linked to myocardin‐associated signaling and maintenance of the differentiated SMCs phenotype, including regulation of SMαA stress fiber formation and contractile phenotype maintenance [40]. Although these studies primarily describe general molecular mechanisms involved in SMCs differentiation rather than direct PBM‐mediated gene regulation, PBM has been shown to modulate several upstream pathways associated with cellular proliferation, mitochondrial activity and redox signaling. Therefore, it may indirectly influence transcriptional programs relevant to SMCs differentiation, although the precise relationship between PBM and SMCs‐specific gene expression remains incompletely understood and requires further investigation [45, 46].

These studies primarily describe established mechanisms governing SMC differentiation and contractile gene expression. Although PBM has been shown to modulate upstream pathways associated with cellular metabolism, proliferation and redox signaling, direct evidence demonstrating PBM‐mediated regulation of myocardin, SRF or other SMC‐specific transcriptional programs during ADSC‐to‐SMC differentiation remains limited. Further studies are required to determine whether PBM directly influences these gene regulatory networks during smooth muscle lineage commitment.

### 2.5 Parameters Influencing PBM Efficiency

The efficiency of PBM is influenced by several critical parameters. Listed in Table 2 below are some key parameters and how they affect PBM efficiency in the differentiation of ADSC into SMCs.

**Table 2:** Key parameters and their influence on PBM efficiency in the differentiation of smooth muscle cells.

Parameters	Influence on PBM efficiency	Refs
1. Wavelength selection	Red light at 600–700 nm and near‐infrared (780–1100 nm) have been reported to enhance ADSC proliferation and support differentiation. 636 nm wavelength increases ADSC viability and proliferation, especially when combined with growth factors. 660 nm wavelength enhances mitochondrial activity and cell proliferation. Yellow and orange wavelengths promote proliferation but do not affect mitochondrial activity significantly.	[25, 45, 47, 48, 49, 50]
2. Energy density (Fluence)	Energy densities (0.5, 1, and 2 J/cm^2^) influence cell viability and proliferation. Demonstrated when PBM used on ADSC applied at 0.5 J/cm^2^ and 635 nm augmented cell viability on the 14th day. Fluence at 5 J/cm^2^ is also critical for effective PBM. PBM applied at 5 J/cm^2^ and 636 nm increased ADSC viability and proliferation within 72 h. No significant influence on differentiation was observed during this period.	[45, 46, 49, 51, 52]
3. Growth factors	Growth factors such as retinoic acid (RA) and TGF‐β play significant roles in ADSC to SMCs differentiation. In combination with PBM, growth factors have been reported to enhance the expression of SMCs‐associated markers. Reported differentiation markers include SMαA and SMMHC.	[53, 54, 55, 56, 57]
4. Co‐culture conditions	The co‐culturing of ADSC with SMCs (1:1 ratio) has been associated with increased ADSC proliferation following PBM exposure. Co‐culture conditions have also been reported to increase SMC‐associated marker expression while reducing stem cell marker expression, indicating enhanced differentiation.	[45, 46]
5. Cell culture conditions	The presence of glucose in the medium greatly impacts PBM efficacy by increasing ATP but decreasing ROS levels. Factors such as serum concentration, cell confluency and oxygen levels may influence cellular responses to PBM.	[58, 59]
6. Time of exposure and exposure conditions	PBM at 636 nm and 5 J/cm^2^ increased ADSC viability and proliferation at 24–72 h post‐irradiation. No significant SMCs differentiation was observed during this period. Stem cell and SMCs marker expressions have been reported to vary with time. Stem cell markers (β1‐integrin and Thy‐1) were expressed 72 h post‐irradiation, whilst the latter marker was downregulated at 48 h, suggesting the influence of the timing of PBM exposure and its influence in the expression of specific differentiation markers.	[49]
7. Reactive oxygen Species	Physiological ROS contributes to ADSC proliferation and differentiation. PBM can modulate intracellular ROS production, therefore influencing signaling pathways associated with ADSC differentiation. Conversely, higher levels of ROS can inhibit self‐renewal.	[25, 60, 61, 62]
8. Mitochondrial Metabolism	Elevated levels of mitochondrial membrane potential (MMP) reflect enhanced mitochondrial activity and have been associated with increased differentiation capacity. PBM, particularly near‐infrared and green irradiation, has been shown to increase MMP, indicating enhanced cellular bioenergetic activity. Increased mitochondrial activity following PBM has also been associated with enhanced ADSC proliferation and migration.	[25, 47]
9. Mechanical strain	Mechanical strain in combination with PBM and growth factors has been reported to enhance SMC differentiation, as demonstrated by increased expression of SMC‐associated markers.	[53, 54, 57]

Table 2 above outlines parameters influencing PBM efficacy during ADSC differentiation toward SMCs. Reported outcomes include effects on cellular metabolism, proliferation/viability and differentiation, which are distinguished where possible to avoid treating these biologically distinct responses as equivalent.

### 2.6 Role of 3D Hydrogel Scaffolds in PBM‐Enhanced Differentiation

3D hydrogels act as a supportive framework in ADSC research, allowing for a biomimetic environment resembling the natural ECM [63]. This environment is conducive for cell survival, proliferation, differentiation and migration, an essential characteristic for TE and regenerative medicine applications [64, 65]. The ability of hydrogels to retain significant amounts of water is advantageous in providing a hydrated environment required for cell viability and proliferation [66]. Certain gelatin‐based hydrogels maintain high cellular biocompatibility and cell adhesion, while arginylglycylaspartic acid (RGD) incorporated hydrogels enhance initial attachment and maintain cell morphology, allowing for efficient adipogenic differentiation [67]. Recent studies suggest that PBM may further enhance the regenerative potential of 3D hydrogel systems by modulating mitochondrial activity, ATP production and cellular proliferation within biomimetic microenvironments. In ADSC spheroid and hydrogel models, PBM has been shown to improve cellular viability, migration and metabolic activity, suggesting a synergistic relationship between hydrogel‐based culture systems and PBM‐mediated cellular responses [52, 68]. Furthermore, the optical and mechanical properties of hydrogels may influence light penetration and distribution, thereby affecting PBM efficiency and subsequent differentiation outcomes.

Moreover, it has been demonstrated that hydrogels with the addition of ECM components such as fibronectin and collagen I enhance ADSC differentiation when compared to the traditional two‐dimensional methods [69]. One of the major advantages of 3D hydrogels is their ability to provide mechanical integrity and can be 3D‐printed to mimic tissue stiffness variations. The variation in density cross‐linking along with the incorporation of various materials makes this possible during printing [70, 71].

### 2.7 Types of Hydrogels Used for ADSC Stem Cell Differentiation

Several polymers used to make hydrogels are from natural sources including alginates, collagen, fibrin, chitosan, gelatin and hyaluronic acid, among many more. These polymers are advantageous due to their biocompatibility, environmental responsiveness and wide availability [63]. Collagen comprises one of the ECM proteins found in mammalian tissue and due to its biocompatibility, versatility and its biodegradability it has been a preferred biomaterial for studies in TE [63]. Collagen type 1‐based hydrogels have been proven to be covalently cross‐linked to protein patterns to promote cell–material interactions [72].

Synthetic polymers are tunable, with customizable mechanical and chemical properties. Moreover, features such as the block structures, molecular weights and the degradable linkages allow for tunable properties and modulation of the mechanical characteristics and degradation rate [70]. Some disadvantages associated with synthetic hydrogels include limited adhesive sites, reduced bioactivity and potentially unfavorable degradation products [70, 73].

Lastly, one of the synthetic hydrogels, polyethylene glycol (PEG), is dependent on the synthesis conditions and molecular weight. It possesses high biocompatibility, cost‐effectiveness and water solubility and as such has received approval from the Food and Drug Administration (FDA) [73]. Despite its strong traits, PEG in its purest form as a hydrogel cannot support cellular adhesion and proliferation, however, with the addition of the peptide RGD, cellular adhesion is promoted thereby enhancing hydrogel performance [73]. Recent studies have suggested that combining PBM with hydrogel‐based culture systems may further enhance ADSC viability, proliferation and differentiation through modulation of cellular metabolism and mitochondrial activity within biomimetic microenvironments [52, 68].

### 2.8 Influence of Hydrogel Properties on PBM Efficacy

PBM efficacy can be significantly influenced by various hydrogel properties such as composition, stiffness and porosity. In one study, varying concentrations of fibrin hydrogels demonstrated different levels of cell viability following PBM exposure, with near‐infrared (NIR) light at 840 nm reported to be the most effective [74]. Mechanical properties such as stiffness may also be altered using light‐responsive systems, thereby influencing cellular behaviour and mechanotransduction pathways [70]. This modulation is advantageous as it allows greater control over the cellular microenvironment, potentially enhancing PBM efficacy and subsequent cellular responses [52, 68].

Porosity is another important hydrogel characteristic as it governs transport properties and influences cell fate. Porous hydrogels improve nutrient and oxygen diffusion, which are important for maintaining cell viability and supporting PBM‐mediated cellular activity [63]. Hydrogel porosity may be regulated through photopolymerization, where ultraviolet or visible light induces the rapid transformation of reactive monomers, oligomers or liquid polymers into solid biomaterials suitable for TE applications [70, 73]. Lastly, the optical clarity provided by hydrogel systems is advantageous since it permits efficient penetration of PBM light into encapsulated cells (Brendon [75]). Therefore, hydrogel composition, stiffness and porosity and clarity are critical factors influencing PBM efficacy in enhancing cell viability, proliferation and overall physiological activity in TE constructs.

### 2.9 Potential Interactions between Hydrogels and PBM in SMCs Differentiation

**Figure 2:**
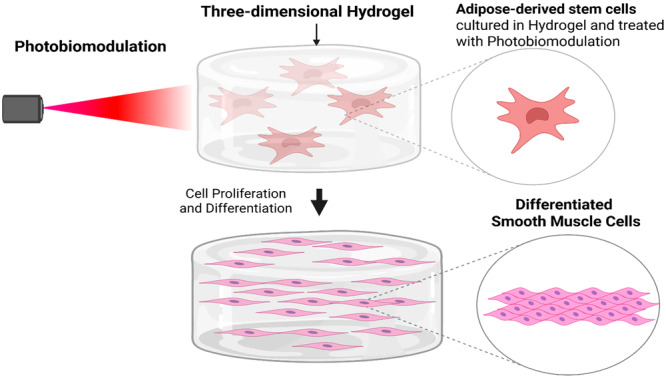
Conceptual diagram illustrates the potential interactions between PBM and 3D hydrogel microenvironments during ADSC differentiation into SMCs. Created with BioRender Christevie Mbuyu. ADSC, adipose‐derived stem cells; PBM, photobiomodulation; SMC, smooth muscle cell.

The combined application of hydrogels and PBM has attracted increasing interest in TE due to their ability to provide biomimetic mechanical and biochemical microenvironments supportive of muscle tissue growth and differentiation. Viscoelastic hydrogels have been associated with improved cellular proliferation and differentiation owing to rapid stress relaxation properties that influence cellular behavior and mechanotransduction pathways [76]. Figure 2 provides a conceptual overview of how hydrogel‐based microenvironments and PBM may collectively influence ADSC differentiation toward SMC phenotypes.

As illustrated in Figure 2, hydrogels provide a biomimetic three‐dimensional environment that supports cellular attachment, proliferation and mechanotransduction [18, 77, 76] while PBM acts as an external stimulus capable of modulating cellular metabolism and signalling pathways [68, 78]. The combined influence of these factors may affect ADSC behaviour and differentiation [52, 68], however, direct evidence demonstrating coordinated hydrogel‐PBM interactions during ADSC‐derived SMC differentiation remains limited. PBM has additionally been associated with modulation of inflammatory responses and nitric oxide synthesis, factors relevant to tissue repair and cellular differentiation [68, 79]. Collectively, these findings suggest that hydrogel properties may influence PBM‐mediated cellular responses through regulation of cellular behavior, mechanotransduction and microenvironmental conditions. However, studies specifically investigating hydrogel–PBM interactions during ADSC‐derived SMCs differentiation remain limited and require further investigation.

### 2.10 Potential Applications of PBM in SMC Differentiation

PBM has shown therapeutic promise, with several studies reporting molecular and cellular effects potentially relevant to SMCs differentiation [80]. PBM‐associated effects reported in MSCs and ADSCs include enhanced proliferation, migration and differentiation‐associated responses following irradiation, although direct evaluation of SMCs‐specific markers such as SMαA and SMMHC remains limited within the currently available literature [80, 81].

Additionally, PBM has been associated with anti‐inflammatory and regenerative effects, including modulation of inflammatory responses and stimulation of pro‐angiogenic and pro‐myogenic factors relevant to tissue repair processes [82]. Collectively, these findings provide indirect evidence suggesting that PBM may influence pathways associated with SMCs differentiation. However, further studies specifically evaluating SMCs‐associated marker expression and optimal PBM parameters are still required to establish direct mechanistic relationships within the context of vascular biology and regenerative medicine.

### 2.11 Challenges for Future Directions

Current PBM protocols for SMCs differentiation face several challenges which may affect successful outcomes. Dose‐dependent effects influence the efficacy of using PBM on SMCs differentiation since it relies greatly on the dose parameters which have been selected such as wavelength, energy density and the duration by which exposure occurs, particularly in a 3D setting. Achieving optimal SMCs differentiation outcomes requires identification of PBM parameters that do not induce adverse effects, which can be challenging [47, 83, 84]. Moreover, inefficient and inconsistent differentiation protocols result in increased variability across various cell lines with low differentiation efficiency, this makes achieving reliable and reproducible results a challenge [85, 86].

Another notable factor that poses a challenge in PBM protocol for optimal SMCs differentiation is the difficulty in controlling the switch between synthetic and contractile phenotypes. Synthetic SMCs are prone to proliferation and ECM production, whereas the contractile phenotype is required for functional vasculature, and existing tissue‐engineering strategies still face challenges in regulating this phenotypic transition [57, 87]. While PBM is known to influence cellular activity, the exact molecular and cellular mechanisms underlying their effect on ADSCs within 3D hydrogel matrices and during SMCs differentiation are not yet fully understood. This gap in knowledge and understanding hinders the development of specific PBM protocols that can consistently achieve targeted differentiation outcomes [88, 89]. Similarly, hydrogels and their diverse properties including composition, stiffness and degradation rates and their influence on the behavior of ADSC under PBM treatment is not well elucidated. Customizing these hydrogel properties to synergize with PBM could significantly improve TE systems [90].

Lastly, regardless of the extensive research being conducted, there is still a scarcity of comprehensive clinical guidelines on the application of PBM for SMCs differentiation. The lack of standardized treatment protocols leads to variations in clinical practices and results, impeding the broad adoption of PBM in therapeutic settings [80]. Overcoming these limitations would require efforts toward the standardization of PBM parameters, enhancement of efficient and consistent protocols, improved understanding of underlying mechanisms and establishment of evidence‐based clinical guidelines that may enhance its effectiveness in promoting SMCs differentiation.

## 3 Conclusion

The convergence of ADSC, PBM, and 3D hydrogel scaffolds offers a promising approach in regenerative medicine and TE [91], particularly with regards to SMCs differentiation. The review highlights PBM's potential and the challenges for clinical translation. ADSCs are advantageous as a source of MSCs since they are abundant and easily accessible whilst still able to secrete bioactive components in the form of cytokines and growth factors. These elements are beneficial for tissue repair, angiogenesis and regeneration, making ADSCs ideal candidates for the TE field specifically in hollow organ and vascular reconstruction and repair. PBM has been shown to modulate cellular mechanisms including mitochondrial activity, ROS production and several signaling pathways such as MAPK/ERK, Wnt/β‐catenin and TGF‐β. These pathways have established roles in cellular proliferation, migration and smooth muscle biology. Whether PBM directly regulates these transcriptional networks during ADSC‐to‐SMC differentiation has yet to be determined. Together, these pathways play varying albeit crucial roles in cell proliferation, migration and differentiation. The non‐invasive nature of PBM along with its ability to regulate gene expression and cellular metabolic activities enhances its therapeutic potential. However, for successful optimization, PBM efficacy relies greatly on certain parameters such as wavelength, energy density and exposure time in combination with growth factors. The major signaling pathways and PBM‐associated parameters influencing these responses are summarized in Tables 1 and 2, respectively. Optimization of outcomes requires consistency and efficiency of these variables.

3D hydrogels also play important roles in achieving differentiation, this is achieved by providing biomimetic environments that closely replicate the ECM and hence encouraging viability, proliferation and differentiation. Additionally, the structural stability and porosity they provide allow for necessary nutrients and oxygen diffusion required for cellular survival. Each type of hydrogel possesses distinct advantages and limitations to different variables. Adapting various factors such as composition, stiffness of hydrogels is fundamental for an environment favorable for ADSC differentiation into SMCs phenotype.

Whilst numerous studies exist highlighting the individual benefits of both PBM and hydrogels, and their respective contributions in ADSC‐related differentiation into SMCs lineage; existing literature on the synergistic effects or its potential thereof, of both PBM and hydrogel on ADSC differentiation into SMCs is very minimal. This may therefore hinder the ability to establish standard protocols for successful reproducibility and consequent clinical application for regenerative medicine. PBM's regulation of ROS levels, mitochondrial activity and inflammatory responses complement the mechanical and biochemical cues offered by hydrogels. These dynamics hold great promise for optimizing SMCs differentiation. Therefore, further experimental studies on the synergistic effects are required to investigate and confirm this hypothesis.

Some challenges that may be encountered to achieve this include the lack of standardized PBM protocols for optimization of parameters; additional research is required to establish optimal parameters of SMCs differentiation. Similarly with hydrogels, while they offer a versatile platform, fine‐tuning certain properties is required to promote effective synergy with PBM. Additionally, ensuring a balanced transition between synthetic and contractile phenotype of SMCs is important for functionality but remains an evident hurdle. Moreover, a more in depth understanding of the molecular mechanisms by which PBM affects ADSC behavior within a 3D hydrogel setting is imperative to further develop therapeutic strategies. Furthermore, the development of consistent and scalable methodologies is necessary for the adaptation of these techniques for clinical application.

Lastly, this integrative approach of ADSCs, PBM, and 3D hydrogel scaffolds offers a means to overcome current limitations existing in SMCs differentiation and TE, offering new solutions for the treatment of SMCs‐related disorders. Future recommendations include addressing existing challenges through focused research and optimization, as well as assessing the long‐term stability and functionality of the differentiated SMCs in vivo will broaden the path for clinical applications and particularly regenerative medicine and thus improving patient outcomes [88, 92].

## Author Contributions

Christevie Mbuyu and Anine Crous contributed to the design of this work. Christevie Mbuyu and Anine Crous contributed to the interpretation of data. Christevie Mbuyu and Anine Crous analyzed the data. Christevie Mbuyu drafted the work. Anine Crous and Heidi Abrahamse revised critically for important intellectual content. All authors read and approved of the final manuscript. All authors agree to be accountable for all aspects of the work in ensuring that questions related to the accuracy or integrity of any part of the work are appropriately investigated and resolved.

## Ethics Statement

The authors have nothing to report.

## Conflicts of Interest

The authors declare no conflicts of interest.

## Data Availability

Data sharing not applicable to this article as no datasets were generated or analysed during the current study.
